# Preventing Disulfide Bond Formation Weakens Non-Covalent Forces among Lysozyme Aggregates

**DOI:** 10.1371/journal.pone.0087012

**Published:** 2014-02-14

**Authors:** Vijay Kumar Ravi, Mohit Goel, Hema Chandra Kotamarthi, Sri Rama Koti Ainavarapu, Rajaram Swaminathan

**Affiliations:** 1 Department of Biotechnology, Indian Institute of Technology Guwahati, Guwahati, Assam, India; 2 Department of Chemical Sciences, Tata Institute of Fundamental Research, Homi Bhabha Road, Mumbai, Maharashtra, India; LAAS-CNRS, France

## Abstract

Nonnative disulfide bonds have been observed among protein aggregates in several diseases like amyotrophic lateral sclerosis, cataract and so on. The molecular mechanism by which formation of such bonds promotes protein aggregation is poorly understood. Here in this work we employ previously well characterized aggregation of hen eggwhite lysozyme (HEWL) at alkaline pH to dissect the molecular role of nonnative disulfide bonds on growth of HEWL aggregates. We employed time-resolved fluorescence anisotropy, atomic force microscopy and single-molecule force spectroscopy to quantify the size, morphology and non-covalent interaction forces among the aggregates, respectively. These measurements were performed under conditions when disulfide bond formation was allowed (*control*) and alternatively when it was prevented by alkylation of free thiols using iodoacetamide. Blocking disulfide bond formation affected growth but not growth kinetics of aggregates which were ∼50% reduced in volume, flatter in vertical dimension and non-fibrillar in comparison to control. Interestingly, single-molecule force spectroscopy data revealed that preventing disulfide bond formation weakened the non-covalent interaction forces among monomers in the aggregate by at least ten fold, thereby stalling their growth and yielding smaller aggregates in comparison to control. We conclude that while constrained protein chain dynamics in *correctly* disulfide bonded amyloidogenic proteins may protect them from venturing into partial folded conformations that can trigger entry into aggregation pathways, *aberrant* disulfide bonds in non-amyloidogenic proteins (like HEWL) on the other hand, may strengthen non-covalent intermolecular forces among monomers and promote their aggregation.

## Introduction

Accumulation of ordered protein aggregates like amyloid is a symptom of several diseases like Alzheimer’s, Parkinson’s and human lysozyme amyloidosis [1]. The causative molecular mechanisms responsible for such diseases are topics of intense research currently. The role of aberrant disulfide bonds in promoting protein aggregation has assumed significance lately owing to observation of intermolecular disulfide bonds among aggregates of mutant Cu Zn superoxide dismutase (SOD1) in the spinal cord of transgenic mice [2] and in paired helical filaments of tau protein existing as neurofibrillary tangles in human brain [3]. The former is implicated in amyotrophic lateral sclerosis while the latter is the hallmark of Alzheimer’s disease. Nonnative disulfide bonds were also among the prominent covalent modifications of crystallins isolated from cataractous lenses [4].

Formation of disulfide bonds has been shown to influence aggregation of proteins in multiple ways. For example, intramolecular disulfide bonds in tau protein can result in conformations that lock up the protein chain such that further aggregation is slowed down. In contrast, intermolecular disulfide bonds in the same protein have been shown to accelerate aggregation [3]. Several instances where formation of disulfide bonds can alter the aggregation pathway [5], reduce toxicity of amyloid fibrils [6] and change material properties of the amyloid fibrils [5] have been reported.

In this work, we investigate the role of disulfide bonds in controlling the aggregation of hen eggwhite lysozyme (HEWL) which has eight cysteine residues per polypeptide chain. Aggregation of HEWL has been well studied in the past [7] serving as model protein. It has been shown previously that zero cysteine mutant of HEWL can form amyloid fibrils at acidic and neutral pH [8]. However, in contrast, presence of DTT has been shown to abolish aggregation in HEWL in alkaline pH [9] by preventing formation of incorrect disulfide bonds. The mechanism by which such disulfide bonds influence aggregation of HEWL at alkaline pH is not clear and needs to be established. Here in this work, we show quantitatively that blocking disulfide bond formationin HEWL a) weakens non-covalent interactions among HEWL monomers in amorphous aggregates considerably as measured by single-molecule force spectroscopy; b) diminishes the size of aggregates as observed by nanosecond fluorescence anisotropy decay and atomic force microscopy (AFM); and c) inhibits amyloid fibril formation as revealed by Thioflavin T (ThT) fluorescence, in stark contrast to unblocked control samples.

## Materials and Methods

### Materials

Sodium dihydrogen phosphate, sodium bicarbonate, sodium thiosulphate, L-cysteine, along with solvents like DMSO, Dimethylformamide (DMF) were purchased from Merck Limited, Mumbai. 8-Anilino-1-naphthalene sulfonic acid ammonium salt (ANS), 2,2′-dithiobis (5-nitropyridine), (DTNP), Hen eggwhite lysozyme (HEWL), iodoacetamide, thioflavin T (ThT) were obtained from Sigma-Aldrich Chemicals Pvt. Ltd., India. 2-dimethyl amino naphthalene 6-sulphonyl chloride (dansyl chloride) was purchased from Invitrogen, USA. AFM Cantilevers PPP-NCL-50 (Point probe Plus/Non- contact/Long cantilever, part 65-262P) was purchased from Molecular Imaging, USA. Muscovite mica, V-1 quality was purchased from Electron Microscopy Sciences, USA.

### Protein Labeling

HEWL was covalently labeled with dansyl chloride following the protocol provided from Molecular Probes with minor modification as reported previously [10]. The HEWL and dansyl concentrations were measured using absorbance at 280 nm and 339 nm, respectively. The protein to dye labeling ratio in the conjugates varied between 1 and 2.

### Sample Preparation and Incubation

Stock solution of HEWL was freshly prepared in deionised water (MilliQ, Millipore, India). For inducing aggregation, this stock was diluted in 50 mM phosphate buffer at pH 12.2 to a final concentration of 70 µM. The samples were incubated at room temperature (22–28°C) and all measurements were performed at 25°C.

### Cysteine Carboxymethylation Reaction by Iodoacetamide

Iodoacetamide was dissolved in DMF to make a stock solution of 1.5 M. 8 mL of this stock solution of iodoacetamide was directly added in four installments of 2 µL each to 1 mL of HEWL sample periodically after completion of 2, 6, 12 and 24 hours of incubation in pH 12.2. No further addition of iodoacetamide was made to HEWL sample thereafter. The above procedure was chosen because previous results had shown that a significant fraction of HEWL disulfide bonds break in pH 12.2 to yield free –SH groups during 2–24 hours after incubation in alkaline pH [9]. Also this procedure is simpler to perform requiring no prior unfolding by guanidine hydrochloride and reduction by DTT. For the progress of reaction, sample was kept at room temperature in dark condition.

The free thiols in HEWL with or without added iodoacetamide in the medium were estimated using DTNP assay described previously [9]. Briefly, DTNP (50 µM) reacts with free cysteine (0–100 µM) at pH 7 to yield a product that absorbs at 387 nm. The unknown concentration of free thiols in HEWL can be determined from the standard plot generated with L-cysteine.

Our experiments with iodoacetamide following the procedure above yielded on average modification of 60–70% of thiols among 8 cysteine residues of every HEWL polypeptide chain at pH 12.2. The remaining thiols were not alkylated probably because they were buried inside the core of HEWL and thus inaccessible to iodoacetamide.

### Steady-state Fluorescence Measurements

All steady-state fluorescence measurements were carried out using Fluoromax-3 spectrofluorometer (Jobin-Yvon Horiba Inc., USA). To minimize the photobleaching, excitation light shutter was kept closed when not recording fluorescence intensity.

#### ANS binding

The stock concentration of ANS (1 mg/mL in deionised water) was measured using extinction coefficient of 4,950 M^−1^ cm^−1^ at 350 nm. Binding of ANS (10 µM) to HEWL (diluted to 5 µM) in pH 12.2 buffer inside a cuvette was monitored from fluorescence of ANS. Steady-state fluorescence emission spectrumof ANS was recorded from 400 to 600 nm after excitation at 380 nm (excitation slitwidth 1 nm and emission slitwidth 10 nm). The background intensity from Raman scatter and buffer were negligible (<5%) compared to sample fluorescence intensity under identical conditions. These were however subtracted from sample emission spectra.

#### ThT binding

Stock solution of ThT was made by dissolving 3 mg in 3 mL of deionised water (MilliQ). This solution was subsequently filtered through 0.45 µm filter. The filtered solution was diluted in ethanol before taking absorbance at 416 nm. The ThT concentration was calculated using extinction coefficient 26,620 M^−1^ cm^−1^. Aliquots of ThT stock solution were stored in −20°C. For fibril binding assays 1 mM ThT was prepared in deionised water. This solution was diluted to 0.5 mM in 20 mM Gly-Gly buffer, pH 8.5. HEWL protein aggregates were diluted to 7 µM and ∼11 µM ThT was added into this protein sample, while sample volume was made up to 1 mL using 20 mM Gly-Gly pH 8.5 buffer. It was seen that fibrils bind strongly with ThT at molar ratio of ∼1∶2 protein to ThT [11]. The spectrum of vortexed sample was taken immediately by exciting at 450 nm (1 nm slit width) and recording emission from 470 to 550 nm with 5 nm slit width. The background fluorescence intensity including Raman scatter was subtracted from sample spectra. The integrated fluorescence intensity of ThT alone measured in absence of HEWL at 48 hours was normalized to unity. The measured integrated fluorescence of all samples were scaled up against this normalized value and plotted to reveal the observed fold increase in ThT fluorescence against incubation time of HEWL at pH 12.2 after averaging independent spectral scans.

### Steady-state Fluorescence Anisotropy Measurements

The steady-state fluorescence anisotropy (*r_ss_*) was measured after G-factor correction and dark counts subtraction as described previously [10]. For this measurement HEWL samples (∼70 µM) contained 2 µM of dansyl-conjugated HEWL while remaining protein was unlabelled. The *r_ss_* of dansyl-conjugated HEWL was monitored after different time intervals of incubation.

Dansyl-conjugated HEWL was excited at 375 nm (slit width = 1 nm) and emission at 440 nm was collected with a slit width of 5 nm. Each measurement was done in duplicate, while data reported are averages of three such measurements. The increase in r_ss_ of dansyl-conjugated HEWL with incubation time in pH 12.2 was fitted to the von Bertalanffy equation below:
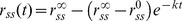
(1)


Here, refers 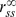 to r_ss_ at t = infinity, while 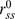 refers same at *t* = 0. *k* denotes the rate constant for increase in anisotropy.

### Time-resolved Fluorescence Measurements

Time-resolved fluorescence intensity and anisotropy (G-factor corrected) measurements were performed in LIFE SPEC II spectrometer (Edinburgh Instruments, Livingston, UK) operating in TCSPC mode, collecting emission decay in 4096 channels using a microchannel plate PMT. Dansyl-conjugated HEWL was excited at 375 nm using EPL-375, picosecond pulsed diode laser with instrument response function (IRF) fwhm ∼150 ps. Dansyl probe fluorescence was collected at 440 nm with a temporal resolution of 24.414 ps/channel. For this measurement HEWL samples (∼70 µM) contained 2 µM of dansyl-conjugated HEWL while remaining protein was unlabeled. Fluorescence lifetime data reported are average of three measurements. Intensity decays were analysed by iterative reconvolution using the Marquardt-Levenberg algorithm to extract lifetimes (τ_i_) and amplitudes (α_i_) as given in equation below.
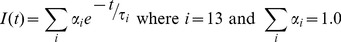
(2)

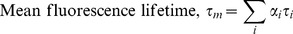
(3)


The raw (G-factor corrected) anisotropy decays were tail-fitted using a sum of two exponentials (equation 4), yielding two rotational correlation times.
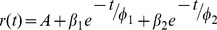
(4)


Here, *A* is a constant dependent on G-factor, β_i_ denotes the amplitude for ϕ_i_, ϕ_1_ and ϕ_2_ refer to the fast and slow rotational correlation times, respectively. The slower rotational correlation time (ϕ_2_) corresponds to global rotational motion of the whole HEWL aggregate. As the 0.15 ns IRF pulse-width is negligibly small in comparison to the time scale of protein rotational motion (>4 ns), the extracted values of ϕ_2_ by this tail-fit approach are not affected by consequences of IRF convolution.

### Atomic Force Microscopy Imaging

The HEWL aggregated samples (10 µL) were dropped on freshly cleaved mica in the presence of 10 mM Mg^2+^ ions and left to adsorb for 2 minutes. The samples were rinsed with 0.2 µm filtered deionized water to remove unadsorbed sample and were dried under nitrogen stream. Samples were imaged in air under AAC MODE (non contact) in PICO PLUS™ AFM purchased from Molecular Imaging, USA. Cantilever type PPP-NCL-50 (resonance frequency, 150 kHz Molecular Imaging) was used for AAC mode. Images were acquired digitally at a scan speed of 1 line/second with 256 data points per line. The AFM images were captured at least three times for every sample condition. The shape and size reported are without tip geometry correction.

### Pulling Molecules Using Atomic Force Microscopy

The force-extension curves were measured using a custom built AFM for pulling single molecules, whose details are given elsewhere [12]. Gold coated cantilevers with silicon nitride tip with spring constants around 40 pN/nm were used for the experiments. In a typical experiment, a small volume (40 µL) of sample solution (120 µM HEWL solution in 50 mM phosphate buffer at pH 12.2) was added to a gold coated coverslip and pulling experiments were performed on it. The pulling speed was maintained at 400 nm/s.

## Results

Our objective in this work was to assess the progress of HEWL aggregation at pH 12.2 under two different conditions. In condition 1, the free –SH groups generated upon exposure of HEWL to pH 12.2 were derivatised by addition of iodoacetamide, to yield S-carboxyamidomethyl cysteine derivative of HEWL which we shall refer to as *thiol-blocked* HEWL. In condition 2, no iodoacetamide was added to HEWL samples at pH 12.2 which we refer to as *control*. Aside from addition of iodoacetamide, HEWL samples in conditions 1 and 2 were identical in all other respects.

### Exposed Hydrophobic Regions Probed by ANS

**Figure 1:**
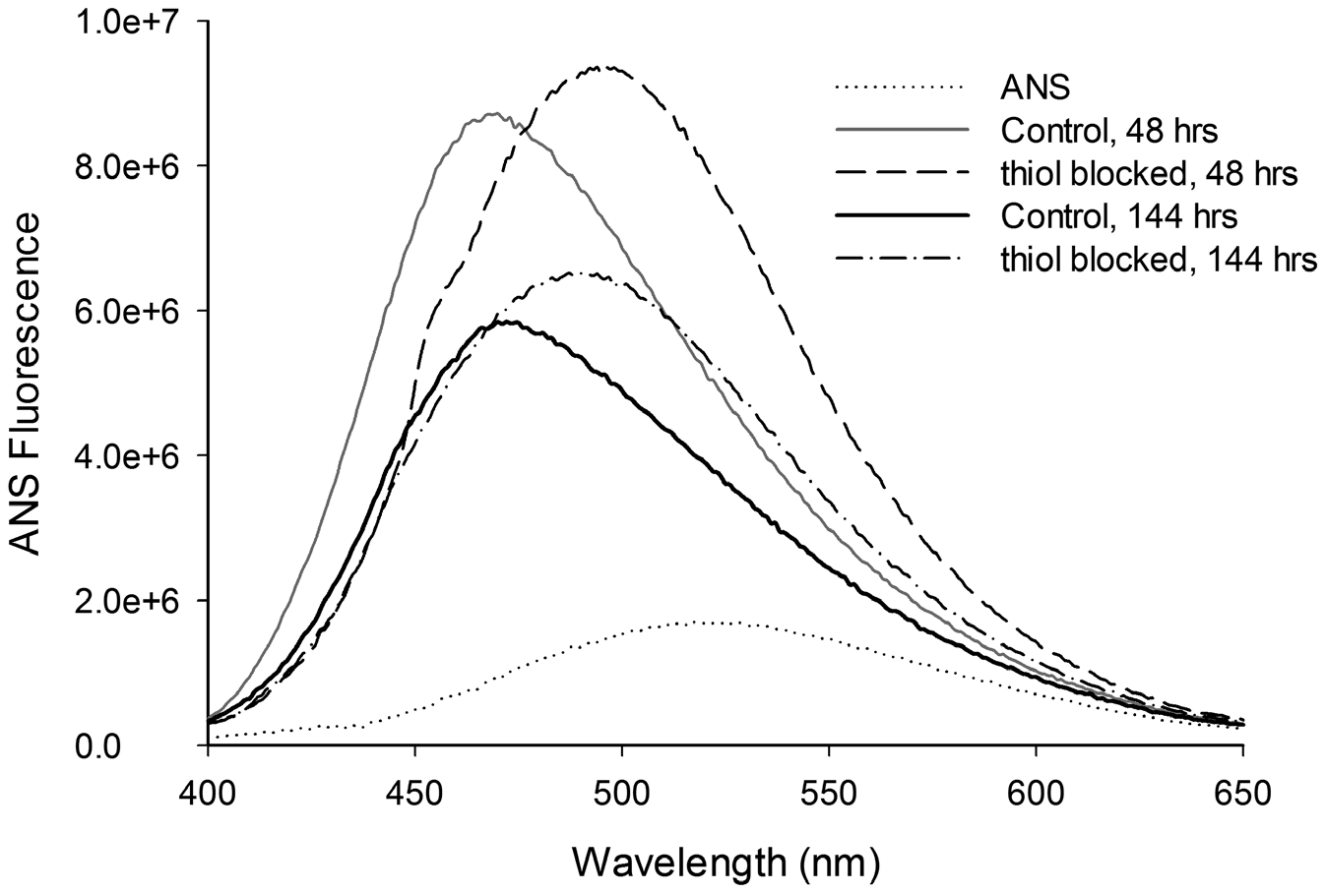
Changes observed in ANS emission spectra in presence of thiol-blocked HEWL and control HEWL samples incubated in pH 12.2 at 301 K for 48 and 144 hours is shown.

Previous work has shown that HEWL on exposure to pH 12.2 becomes partially unfolded initiating the process of aggregation [9], [13]. Exposed hydrophobic regions among the aggregates were monitored using ANS, which serves as an indicator for the growth of aggregates and its suppression [14]. Here we measured extent of exposure among hydrophobic pockets in S-carboxyamidomethyl cysteine derivative of HEWL along with unmodified HEWL after 48 and 144 hours of incubation in pH 12.2 (**Figure 1**). At 48 hours of incubation time both S-carboxyamidomethyl cysteine derivative of HEWL and unmodified HEWL displayed enhanced ANS fluorescence intensity and blue shifted λ_max_ of 498 and 471 nm, respectively compared to ANS alone. At 144 hours, the –SH blocked and unblocked HEWL bound ANS fluorescence intensity decreased substantially while emission λ_max_ of ANS was nearly same as recorded for 48 hours of incubation time. ANS samples (10 µM) in absence of protein displays subdued fluorescence intensity and emission λ_max_∼526 nm in pH 12.2 buffer. The larger area below spectra observed with thiol-blocked HEWL samples in comparison to unblocked samples demonstrate that thiol-blocked HEWL harbors more bound ANS population. The ANS peak shift from 526 nm to 498 nm indicates that ANS molecules are buried inside moderately exposed hydrophobic interior of –SH blocked HEWL [15]. Thus control samples possess deeply buried, more non-polar environment for bound ANS compared to thiol-blocked samples. Evidently blocking thiol groups in HEWL causes the aggregates to be more open and solvent exposed compared to unblocked HEWL samples owing perhaps to absence of cross-linking disulfide bonds.

### Presence of Amyloid Fibrils from Thioflavin T

**Figure 2:**
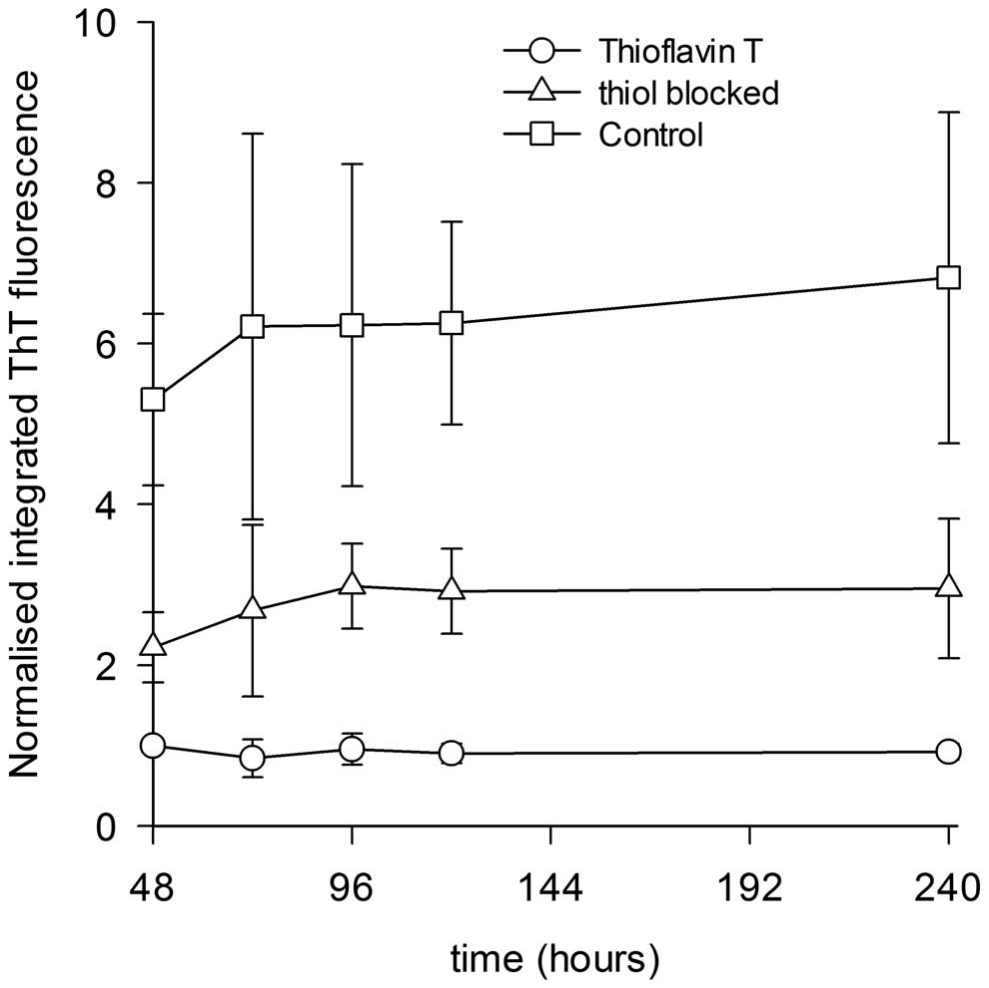
Thioflavin T fluorescence observed in thiol-blocked HEWL and control HEWL samples incubated in pH 12.2 at 301 K for different durations as indicated in X axis is displayed. The error bars represent standard deviations of averaged data from three separate experiments.

Formation of amyloid fibril represents the end point of protein aggregation. It is worthwhile to know if the S-carboxyamidomethyl cysteine derivative of HEWL forms only intermediates like oligomers during incubation at pH 12.2 or does it also form amyloid fibrils? To answer this question, ThT assay of HEWL S-carboxyamidomethyl cysteine derivative was performed after 48 hours. Previous studies have shown that detectable increase in ThT fluorescence in HEWL commences only after 48 hours [7], [9]. At 48 hours, ThT fluorescence intensity from cysteine methylated HEWL was more than 50% diminished compared to control HEWL pH 12.2 samples (**Figure 2**). After a gentle rise till 96 hours, this trend is seen maintained steady till 240 hours. This suggests a significant reduction of amyloid fibril population in thiol-blocked samples compared to control. Residual ThT fluorescence in thiol-blocked samples may arise from ThT binding to oligomers or amorphous aggregates in sample [16]–[18]. However, the fluorescence quantum yield of ThT in such cases is much lower compared to amyloid fibril bound ThT [18]. It has also been reported that positive charge of ThT can interact with negative charge of protein molecule (in this case HEWL whose pI is ∼11.3) in absence of fibrils [19].

The high standard deviations (error bars) noticed among ThT fluorescence data points in presence of HEWL (Fig. 2) has been observed previously too [9]. It indicates a heterogeneous population of oligomers and fibrils among all HEWL samples.

Thus, the ThT fluorescence data clearly argue for a diminished amyloid fibril and oligomer population among thiol-blocked HEWL samples in comparison to control HEWL samples.

### Size and Growth Kinetics of Oligomeric HEWL Aggregates

The growth of HEWL aggregates was monitored by measuring the Brownian rotational motion of fluorescently labeled aggregates using steady state and time-resolved fluorescence anisotropy. For this purpose, monomeric HEWL was labeled with dansyl chloride (an amine reactive fluorescent naphthalene derivative). Dansyl probe-conjugated to protein has a long fluorescence lifetime (>10 ns) which provides an ideal time window of 0–100 ns to monitor the slow rotational motion of large protein aggregates that reveals their size [10], [20]. Previous work by us has shown that dansyl probe has no role in promoting aggregation of HEWL [9].

**Figure 3:**
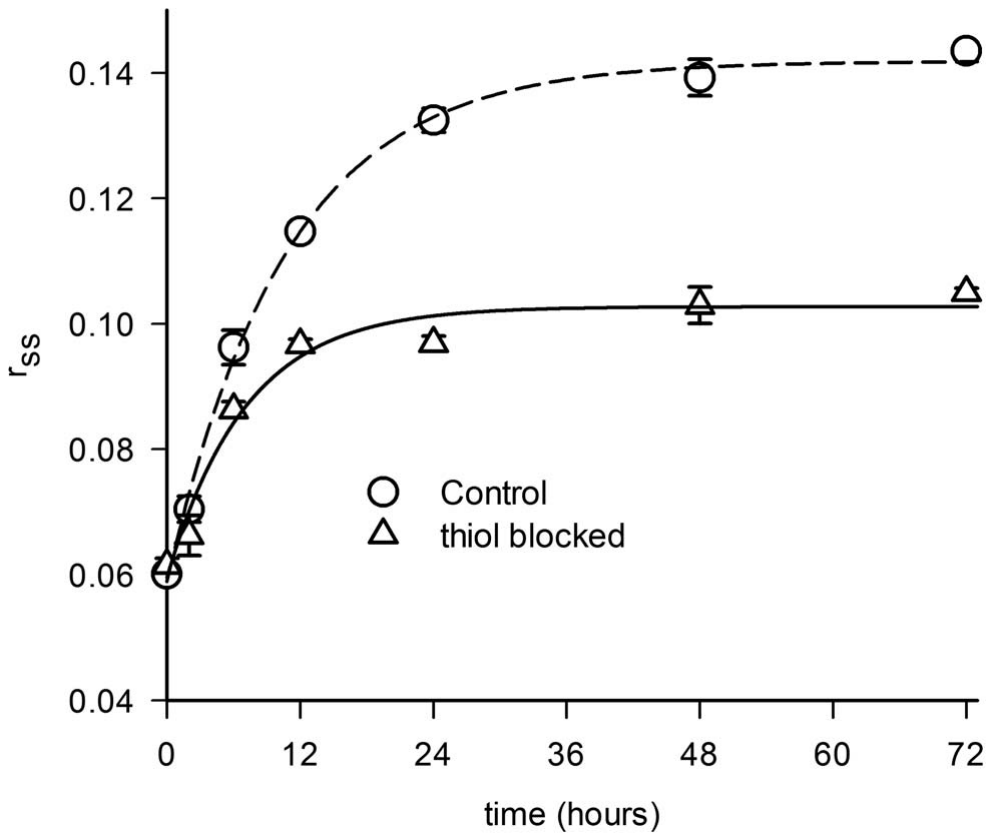
Change in steady state fluorescence anisotropy (r_ss_) of dansyl conjugated thiol-blocked HEWL and dansyl-HEWL (control) sample incubated in pH 12.2 for different durations is shown. The error bars represent standard deviations of averaged data from at least two separate experiments. Each plot was fitted using Eq. 1. The extracted parameters from the fit are displayed in Table 1.

**Figure 4:**
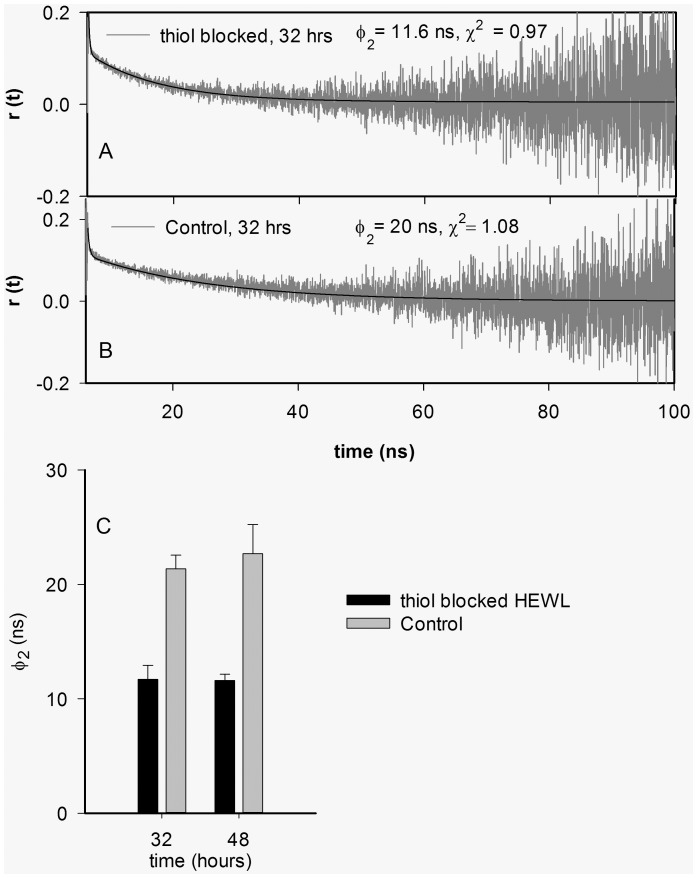
A) Fluorescence anisotropy decay of control dansyl-HEWL in pH 12.2 after 32 hours B) Fluorescence anisotropy decay of –SH blocked dansyl-HEWL in pH 12.2 after 32 hours. C) Bar chart revealing the average global rotational correlation time (ϕ_2_) of thiol-blocked dansyl-HEWL and control dansyl-HEWL after 32 and 48 hours. The error bars represent standard deviations of averaged data from at least two separate experiments.

The growth of thiol-blocked and control HEWL aggregates in pH 12.2 was monitored from the changes in measured steady-state fluorescence anisotropy, r_ss_ of labeled dansyl probe against time (**Figure 3**). While both the r_ss_ traces reveal a rise followed by saturation after 24 hours of incubation in pH 12.2, the anisotropy of thiol-blocked samples saturates at lower r_ss_ values in comparison to control. The mean fluorescence lifetime (τ_m_, Eq. 3) of the dansyl-conjugated HEWL under both control and thiol-blocked conditions remained fairly constant for the duration of experiment (Figure S1, Figure S2 and Table S1) revealing no change in fluorescence quantum yield thereby suggesting that rise in r_ss_ is directly linked to increase in volume of dansyl-conjugated HEWL arising from growth of HEWL aggregates as demonstrated previously too [10]. The traces were fitted to a monomer population restricted growth model using the von Bertalanffy equation (Eq. 1). The values of fitted parameters are listed in **Table 1**. It is observed that growth rate constant *k* is not significantly different between thiol-blocked and control conditions, however the saturation value of r_ss_ (r_ss_^∞^) is significantly less in thiol-blocked condition in comparison to control. This indicates that size of HEWL aggregates formed after 48 hours under thiol-blocked condition is smaller in comparison to control conditions. However, this needs to be confirmed from nanosecond time-resolved fluorescence anisotropy measurements. The typical nanosecond time-resolved fluorescence anisotropy traces of dansyl-conjugated HEWL aggregates incubated for 32 hours in pH 12.2 under thiol-blocked and control conditions along with fits (as per Eq. 4) are shown in **Figures 4A and 4B** respectively. Figure 4C reveals the average value of rotational correlation time component (ϕ_2_) corresponding to global tumbling motion of whole aggregate assembly observed after different times of incubation in pH 12.2. It is evident that HEWL aggregates formed under thiol-blocked condition show a nearly two-fold faster rotational motion (lower ϕ_2_) corresponding to a significantly smaller hydrodynamic volume (from Stokes-Einstein equation) in comparison to HEWL aggregates under control condition at both 32 and 48 hour periods. This reaffirms the earlier conclusion reached from r_ss_ data. Hence the effect of thiol-blocking on arresting the overall growth and assembly of HEWL aggregates is clearly established from the fluorescence anisotropy measurements.

**Table 1:** Parameters extracted (using eq. 1) from fits for growth in steady state anisotropy (r_ss_) shown in Figure 3.

HEWL condition in pH 12.2	r_ss_^∞^	r_ss_^0^	*k* (hour^−1^)	R^2^
Thiol-blocked	0.10	0.06	0.14±0.03	0.97
Control	0.14	0.06	0.09±0.01	1.0

### Pulling Apart HEWL Aggregates by single-molecule Force Spectroscopy

**Figure 5:**
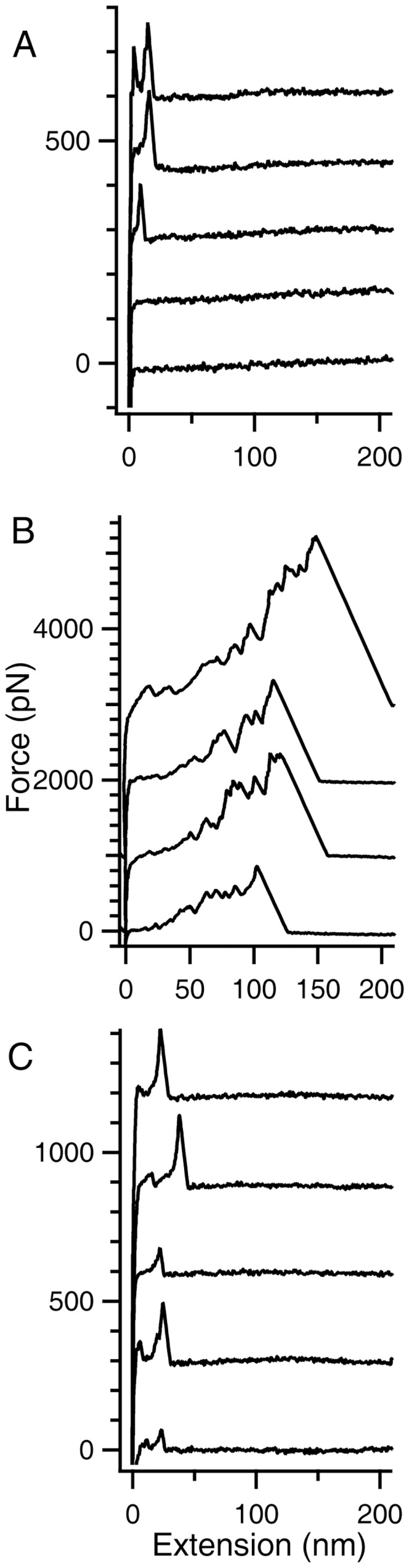
Force-versus-extension (FX) traces of HEWL aggregates from single-molecule force spectroscopy. **A)** FX traces obtained by repeated approach and retraction of AFM cantilever tip onto the gold coverslip on which 40 µl of *freshly prepared* HEWL at pH 12.2 was added. Each trace is offset by 150 pN, with respect to the previous trace. **B)** Representative FX traces obtained by pulling HEWL aggregates formed after 4 days of incubation at pH 12.2. Each trace is offset by 1000 pN with respect to the previous one. See Figure S3 for more traces under this condition. **C)** FX traces of HEWL at pH 12.2 incubated with iodoacetamide at a final concentration of 12 mM for 3 days.

The strength of association between different monomers in the HEWL aggregate is an important parameter to gauge the robustness of aggregate. Measuring this strength for HEWL aggregates formed under the thiol-blocked and control conditions will shed light on the criticality of disulfide bonds in the assembly of HEWL aggregates. Single-molecule force spectroscopy was therefore employed to ascertain the robustness of HEWL aggregates after thiol-blocking. The force-versus-extension (FX) traces of un-aggregated (A), aggregated HEWL at pH 12.2 (B), and aggregated HEWL at pH 12.2 after thiol-blocking (C) were obtained using single-molecule atomic force microscope (SM-AFM) as shown in **Figure 5**.

**Figure 6:**
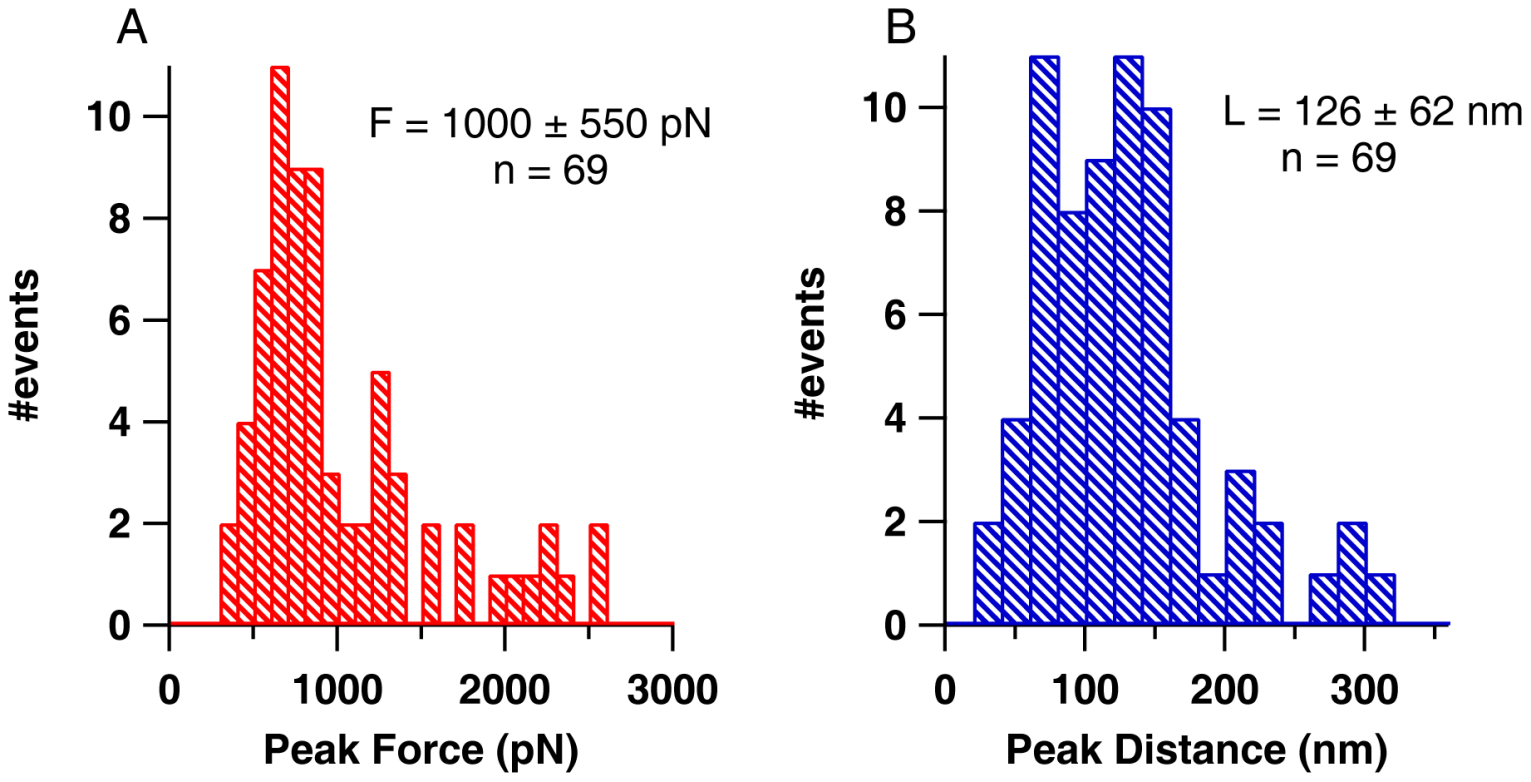
The observed distributions of detachment peak forces (A) and their distances (B) from FX traces of lysozyme aggregates acquired under conditions similar to that described in Figure 5B. The average detachment force of lysozyme aggregates was 1000±550 pN and the average distance at which detachment occurred was 126±62 nm as measured from 69 different FX traces.

- The FX traces clearly indicate that very often there is no molecule adsorbed between the cantilever tip and the gold surface due to the small sizeof protein compared to larger tip diameter (∼20–30 nm). Occasionally low force peaks were observed at the beginning of FX traces, which couldbe due to either unfolding of a single HEWL molecule or any non-specific interaction between cantilever tip and gold surface. Clearly no aggregates are found under this condition.
- In contrast to the case A, here there are large aggregates picked by the cantilever in FX traces. Multiple force peaks in the FX traces are due to the forced dissociation of very strong intermolecular interactions in these aggregates. These traces clearly indicate the varying lengths and mechanical stabilities of these aggregates (300–2600 pN) as evident in **Figure 6** from the observed distributions of detachment peak forces (A) and detachment lengths (B). Force peaks in these FX traces could be attributed to the unraveling of HEWL molecules in the aggregate. It must be noted that the inter-molecular disulfide bonds are still intact in the aggregate. It was earlier shown that disulfide bonds do not rupture below 2 nN [21], [22].
- The FX traces resemble those of freshly dissolved HEWL indicating the absence of any large aggregates in the solution. Iodoacetamide blocks the free –SH groups in HEWL preventing it from forming aggregates.

### Morphology of Aggregates

**Figure 7:**
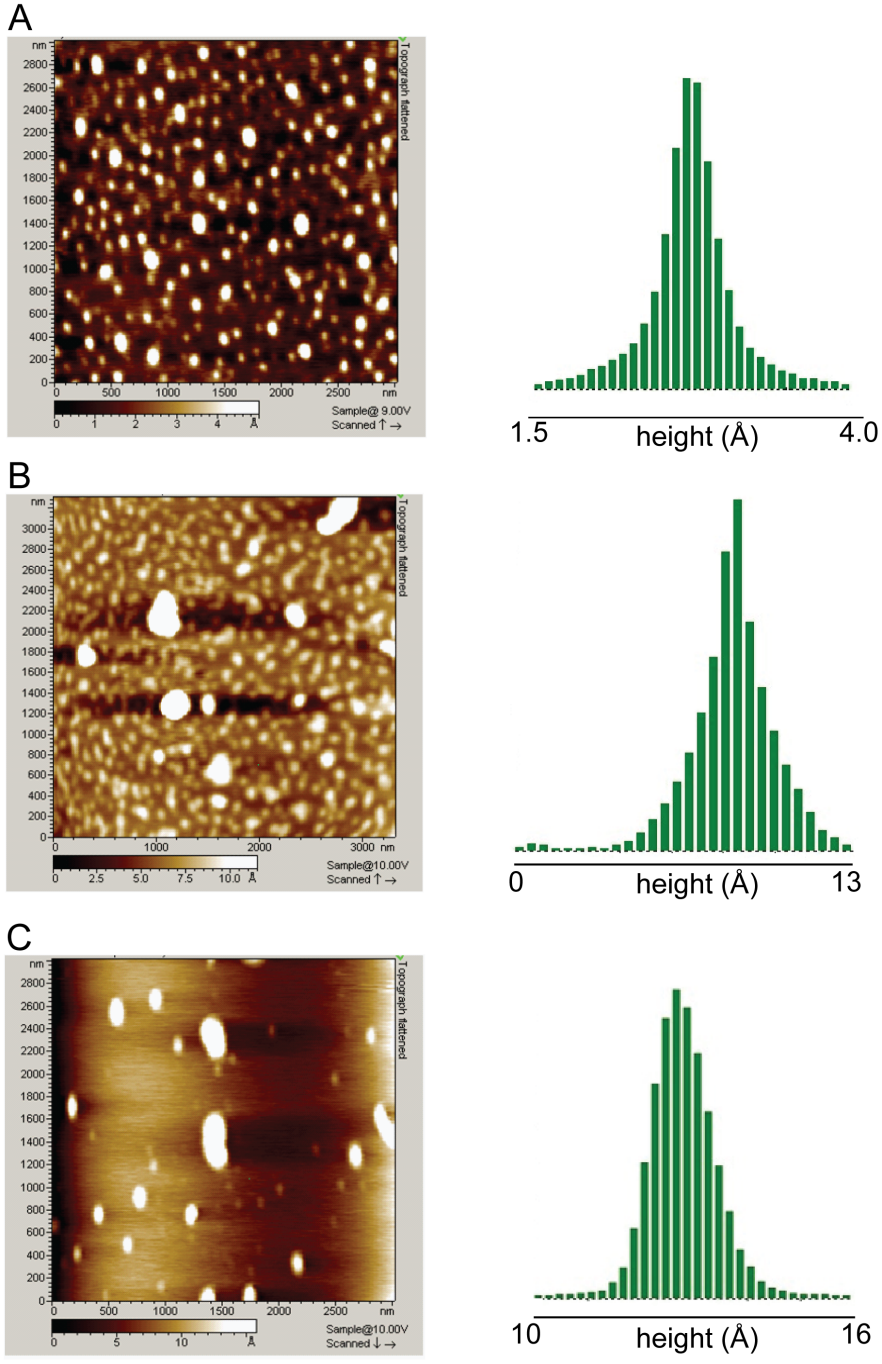
AFM images depicting topography (*left*) and their height distribution in Angstroms (right) for A) thiol-blocked HEWL in pH 12.2 after 72 hours, B) thiol-blocked HEWL in pH 12.2 after 11 days, C) control HEWL in pH 12.2 after 8 days. For height distribution, the threshold employed is identical to the leftmost value on the axis. The heights observed for maximum population in each image were approximately as follows: A) 2.7 Å B) 8.5 Å C) 12.7 Å. Note the reduced height (flatness) of thiol-blocked samples in comparison to control.

After monitoring for hydrophobicity, presence of fibril, changes in size and mechanical strength of aggregates, it was essential to observe their morphology. For this purpose AFM was employed. The AFM images were acquired after incubation of 70 µM thiol-blocked HEWL and 70 µM unblocked HEWL for various durations in pH 12.2. Topography images of thiol-blocked HEWL captured at 72 h (**Figure 7A** **)** reveal predominantly small globular aggregates in large population. At longer incubation times (11 days), few larger aggregates of thiol-blocked HEWL are also visible in a sea of smaller aggregates (**Figure 7B**). The morphology of free thiol containing HEWL (control) revealed a mixture of large elongated and small globular aggregates (**Figure 7C**). The distribution of measured heights (along Z axis) on the right column in Figure 7 clearly shows that thiol-blocked HEWL samples possess shorter heights in comparison to control samples. Thus thiol-modified HEWL aggregates appear more flat (probably owing to less molecular packing) compared to control samples. Interestingly, fibrils were never detected among thiol-blocked HEWL samples even after several weeks while earlier work has shown that 75 µM HEWL (control) formed matured fibrils in pH 12.2 as revealed by AFM [7]. This substantiates ThT fluorescence data which revealed diminished fluorescence in thiol-blocked samples compared to control.

## Discussion

In previous work it was shown that presence of DTT from the beginning in the incubated HEWL sample at pH 12.2 significantly abolished the growth of aggregates [9]. In presence of DTT when all exposed disulfide bonds are reduced, HEWL chain has freedom to sample a larger dynamic ensemble of conformation(s). Perhaps such conformation(s) do not favour the same aggregation pathway as in absence of DTT. For this reason iodoacetamide was added two hours after initiation of aggregation when aggregation competent conformations are active and aggregation is progressing. This way it was ensured that same aggregation pathway was chosen while the role of intermolecular disulfide bonds on aggregate growth can be investigated.

The results show that preventing disulfide bond formation in HEWL at pH 12.2 has significant consequences on the structure and aggregation propensity of the protein. Blocking thiol groups causes non-polar groups in HEWL aggregates to be more solvent exposed and opened up (Fig. 1) probably owing to absence of disulfide bonds that fasten the monomers together causing poor packing and compaction. Also a smaller aggregate (see below) arising from weak intermolecular interactions leading to poor molecular packing can result in poor shielding of hydrophobic groups. Intriguingly, the increased flexibility in the protein conformation(s) under the thiol-modified state apparently does *NOT* affect addition of monomers leading to growth of aggregates as revealed by similar aggregate growth kinetics between control and thiol-blocked samples (Fig. 3). However, the aggregates formed in the thiol-blocked condition are not able to retain the monomers together perhaps due to weak intermolecular forces coupled with absence of cross-linking intermolecular disulfide bonds, leading to their dissociation. This might explain why a) size of aggregates are smaller (Fig. 4) after thiol modification; and b) ordered aggregates like amyloid fibrils are not seen in same condition (Fig. 2). The occasional large aggregate seen in AFM images (Fig. 7) might be attributed to trace amounts of –SH groups that may have escaped modification while the predominantly small flat aggregates in the same images might represent the limiting threshold size of aggregates in absence of disulfide bonds. That the hydrophobic interaction forces keeping the monomers together in these small aggregates in the absence of disulfide bonds are significantly weaker in comparison to control samples is corroborated by the force spectroscopy measurements (Fig. 5).

AFM based force spectroscopy has been used to study the interactions between aggregating proteins like amyloid β [23], [24], alpha synuclein and lysozyme [25]. AFM is not only useful for imaging of aggregates to measure their size and kinetics of aggregation process, but it can also be used to measure the intra- and intermolecular interactions at the single-molecule level [26], [27]. AFM based force spectroscopy has earlier been used to study misfolding and aggregation of synuclein and amyloid beta peptides [27], [28]. Force spectroscopy directly measures the interaction forces between molecules by picking individual aggregates and mechanically stretching them at a constant velocity to rupture the stabilizing intermolecular interactions. The constant-velocity experiment provides the mechanical rupture events in the form of a force-versus-extension trace (FX). The rupture forces measured in the FX traces directly quantify the strength of the intermolecular association in the aggregates. Covalent interactions require typically many nanonewton rupture forces whereas the non-covalent interactions require very low rupture forces.

**Figure 8:**
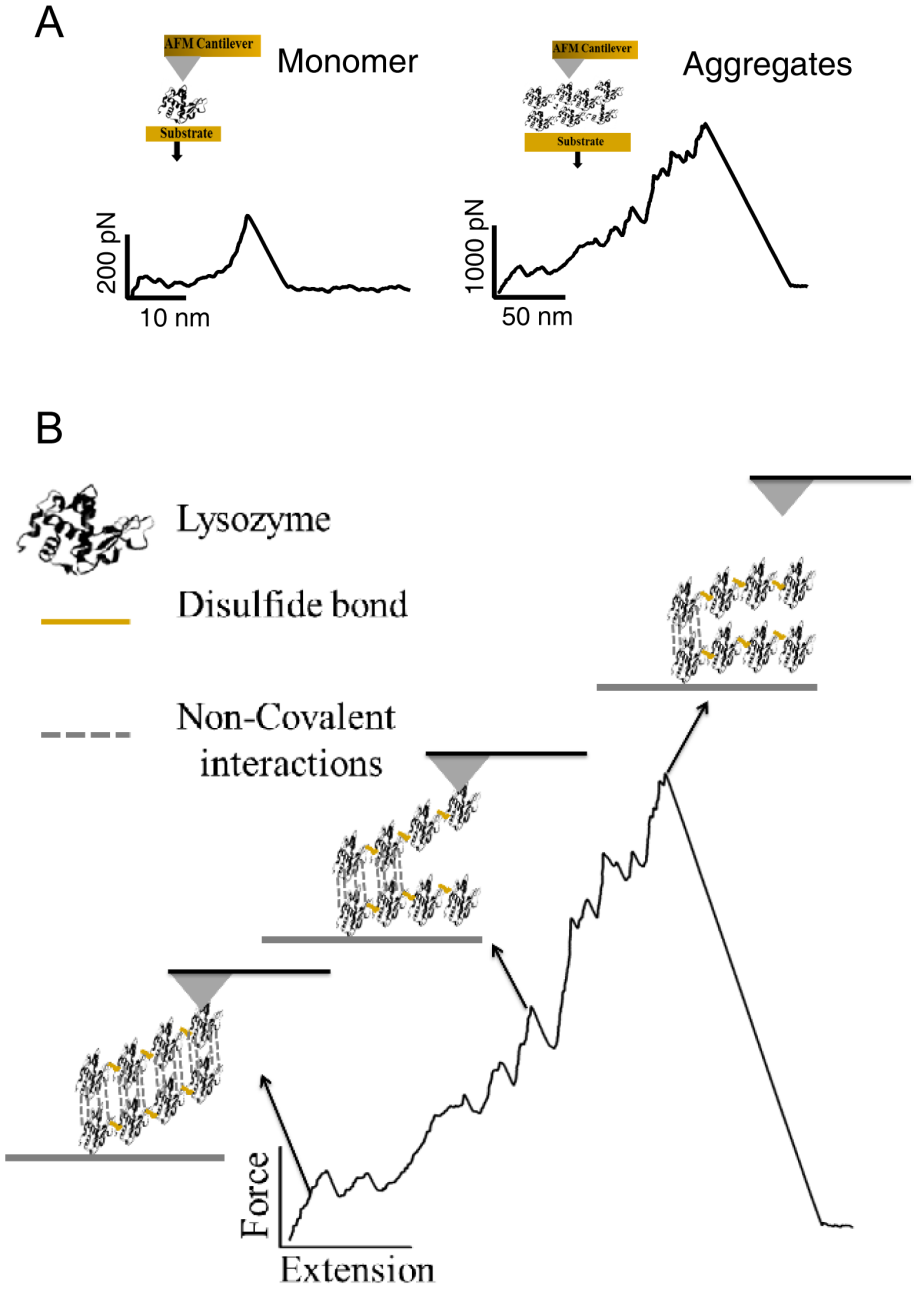
Rupture of HEWL aggregates. (A) The difference in the magnitude of forces needed to mechanically unfold a HEWL Monomer as compared to pulling apart monomers in Aggregates is highlighted. The force-extension profiles observed for thiol-modified HEWL aggregates closely resemble those for the Monomer. (B) Molecular events that occur at different stages during the pulling of aggregates is shown. The non-covalent interactions appear as the weakest link between the monomers which are otherwise cross-linked by intermolecular disulfide bonds in the aggregate.

Previous results of protein-protein interactions on lysozyme molecules in aggregating acidic conditions revealed a maximum force of ∼1100 pN at pH 3 that was roughly ten times more than force encountered under non-aggregating condition at pH 7 [25]. Here we have performed the pulling experiments on lysozyme at pH12.2. The non-aggregated molecules (freshly exposed to pH 12.2) are rarely picked up (due to their small size) and even if they are picked up occasionally they tend to extend and detach at very low forces (Figure 5A and **Figure 8A** (Monomer)). On the contrary, the aggregated molecules (on day 4), owing to their large size, attach frequently between cantilever and the gold surface and have a very high mechanical stability (Figure 5B and **Figure 8A** (Aggregates)). The recorded FX traces suggest that the aggregates unravel in piece-wise manner where individual monomeric units or monomer clusters detach themselves from the aggregate at very high forces varying from 500–2000 pN (Figure 8B) after disruption of non-covalent interactions. It is likely that the cantilever may not be picking up the aggregate as a whole but only a part of it (**Figure 8B**) as suggested by the varying unraveling patterns of the aggregates (Figure 5B and Figure S3) as well as the final detachment length (Figure 6B). The interaction between the individual units in these aggregates is stronger than those formed in the acidic conditions [25]as evident from their higher dissociation forces although a similar ten-fold drop in force is observed in our case too under non-aggregating thiol-blocked condition. It is worthwhile to note that detachment forces of monomer or monomer cluster are <1 nN, implying that it is not the disulfide bonds but the weaker non-covalent hydrophobic interactions that are breaking first (Figure 8B).

To confirm that we have picked large aggregates of lysozyme on day 4, we have also done a pulling experiment on a thiol-blocked sample of lysozyme containing iodoacetamide on day 3, where iodoacetamide blocks the free-SH groups and inhibits disulfide bond based aggregation process. This thiol-blocked HEWL sample showed no pick-ups of molecule or occasionally a small peak indicating the absence of any aggregates. This reaffirms our earlier conclusion that absence of disulfide bonds in HEWL significantly weakens non-covalent interactions such as hydrophobic interactions among monomers in the aggregate preventing further growth and propagation of aggregation.

The results from our work establish that incorrect intermolecular disulfide bonds promote aggregation by a) strengthening hydrophobic interactions among monomers in the aggregate and b) crosslinking HEWL monomers in the aggregate assembly. Presence of similar disulfide bonds among mutant SOD1 aggregates in the spinal cord of ALS model transgenic mice has been observed highlighting their physiological relevance [2]. Xie and coworkers report that HEWL can self-assemble into granular aggregates and subsequently globule-like aggregates at pH 7 and 9 under continuousUV illumination. They show that UV illumination reduces disulfide bonds photochemically causing partial unfolding of HEWL that exposes hydrophobic residues triggering aggregation into granules. On longer illumination and prolonged incubation the granules grow into large globular aggregates facilitated by intermolecular disulfide bonds [29]. Thus mechanism of growth of large HEWL aggregates in their case is identical to what we observe at alkaline pH. In a similar work they also show that fibril formation in HEWL under native conditions can be triggered by photochemical reduction of disulfide bonds [30]. This photochemical approach has been further employed recently to self-assemble bovine α-lactalbumin and doxorubicin into nanoparticles [31]. Lee and Eisenberg have shown that recombinant hamster prion protein (PrP^C^) is converted to an oligomeric, β-sheet rich, fibril formation competent second form PrP^RDX^ by formation of intermolecular disulfide bond. Blocking free thiol by iodoacetamide was shown to prevent this conversion partially [32].

In contrast to aggregation promoting tendency of incorrectly formed disulfide bonds mentioned so far, correctly positioned disulfide bonds in extracellular proteins like insulin and islet amyloid polypeptide avoid aggregation-prone loops in these amyloidogenic proteins from venturing into partial folded conformations that can triggerentry into aggregation pathways [33]. For example it has been argued that disulfide bonds inhibit aggregation of human lysozyme by stabilizing the folded state through reduction in entropy. It has been also argued that when partially folded states are populated, disulfide bonds cause formation of fibrils that are of significantly less toxic nature compared to disulfide reduced fibrils [34]. In another work, it was shown that disulfide bond crosslinked variant of SRC homology 3 domain (SH3) of p85α subunit of bovine phosphatidyl-inositol-3′-kinase (PI3-SH3) domain is more stable, folds faster, aggregates slower, and forms conformationally and functionally different amyloid fibrils than the wild-type domain [35].

## Conclusions

In conclusion, we report that incorrectly formed disulfide bonds actively promote the growth of protein aggregates by strengthening non-covalent intermolecular forces, which are otherwise weak in their absence. This is in contrast to correctly formed disulfide bonds in extracellular proteins which protect them from venturing into aggregation prone pathways. Our findings may lead to a better understanding of the role of aberrant disulfide bonds in diseases like Alzheimer’s and amyotrophic lateral sclerosis.

## Supporting Information

Figure S1 **Fluorescence intensity decay traces of thiol-blocked dansyl-HEWL at different incubation periods in pH 12.2 are shown.**
(PDF)

Figure S2 **Those for control dansyl-HEWL samples are shown.**
(PDF)

Figure S3 **More traces on the rupture of large HEWL aggregates at pH 12.2 are shown.**
(PDF)

Table S1 **Fitted decay parameters are displayed.**
(PDF)
