# Supplementary material for: Preventing Disulfide Bond Formation Weakens Non-Covalent Forces among Lysozyme Aggregates
Source: PLoS One. 2014 Feb 14;9(2):e87012. doi: 10.1371/journal.pone.0087012 (PMC3925087; doi:10.1371/journal.pone.0087012)

Figure S2: Fluorescence intensity decay of *control* dansyl-HEWL samples in pH 12.2 after an incubation period of 1, 6, 12 and 48 hours are shown below.

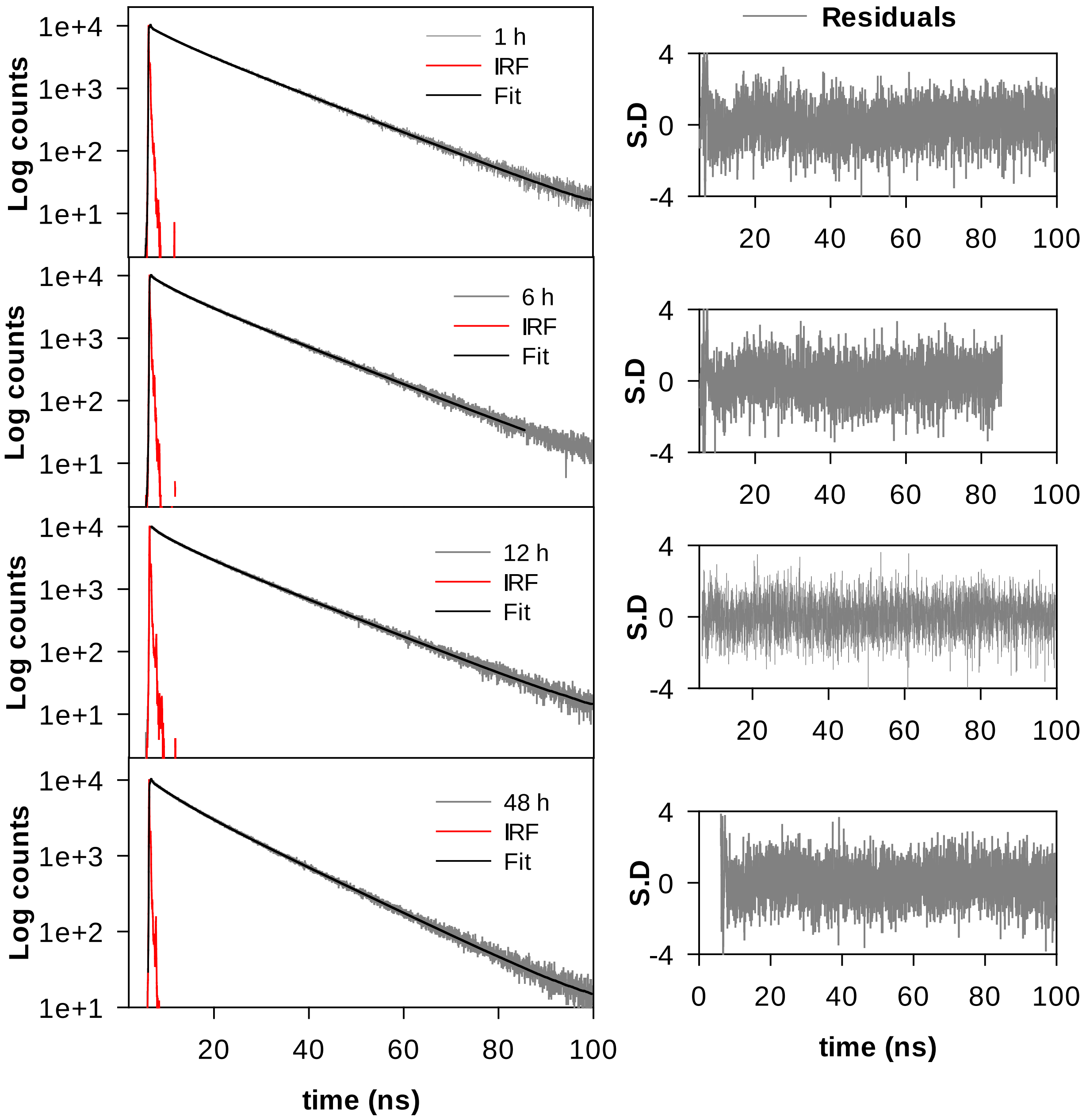

Supplement: Figure S2 — Those for control dansyl-HEWL samples are shown. (PDF) [file pone.0087012.s002.pdf]
