# Supplementary material for: Preventing Disulfide Bond Formation Weakens Non-Covalent Forces among Lysozyme Aggregates
Source: PLoS One. 2014 Feb 14;9(2):e87012. doi: 10.1371/journal.pone.0087012 (PMC3925087; doi:10.1371/journal.pone.0087012)

Figure S3: Additional FX traces of lysozyme aggregates (conditions are similar to those of Fig 5B). The traces are offset by 1000 pN along y-axis. For clarity, traces are shown in black and grey.

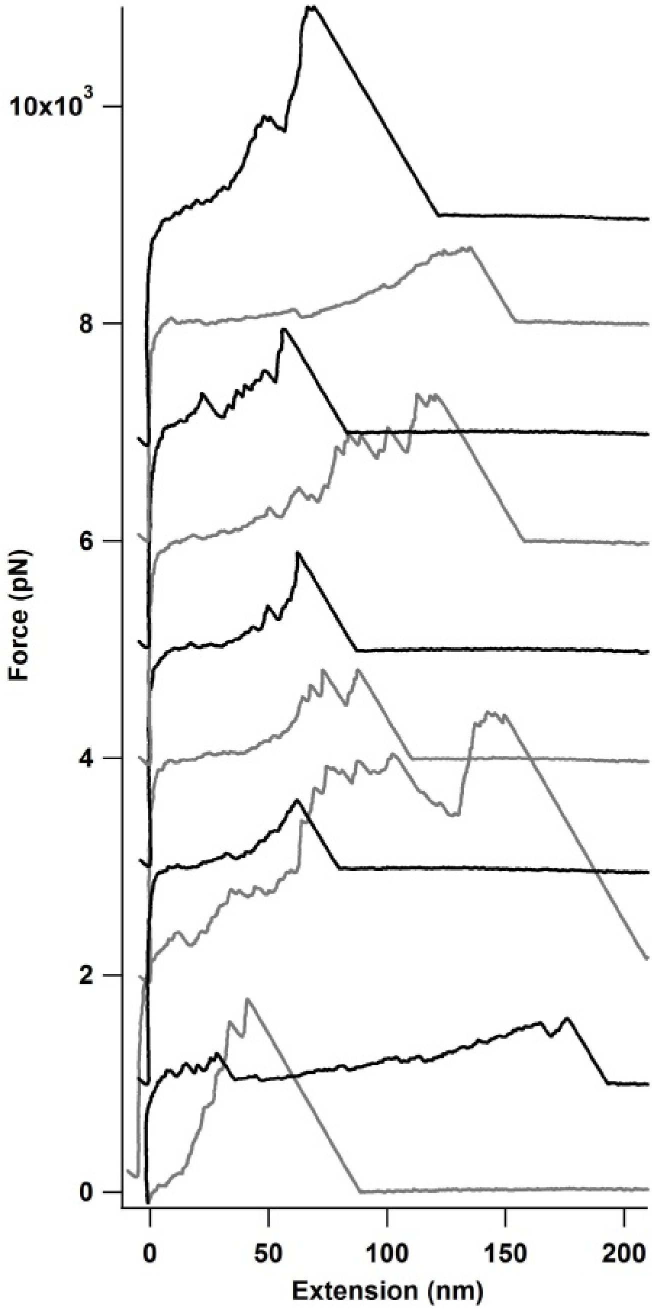

Supplement: Figure S3 — More traces on the rupture of large HEWL aggregates at pH 12.2 are shown. (PDF) [file pone.0087012.s003.pdf]
