# Supplementary material for: Preventing Disulfide Bond Formation Weakens Non-Covalent Forces among Lysozyme Aggregates
Source: PLoS One. 2014 Feb 14;9(2):e87012. doi: 10.1371/journal.pone.0087012 (PMC3925087; doi:10.1371/journal.pone.0087012)
Supplement: Table S1 — Fitted decay parameters are displayed. (PDF) [file pone.0087012.s004.pdf]

Table S1: Fluorescence lifetime parameters of dansyl-HEWL samples extracted from fluorescence intensity decay analysis. See Figures S1 and S2 for the fits and residuals.

Condition	Time (hours)	τ_1 (ns)	α_1	τ_2 (ns)	α_2	τ_3 (ns)	α_3	$\square_m$ (ns)	χ^2
Thiol-blocked HEWL, pH 12.2	1	0.17	0.02	4.89	0.08	14.74	0.90	**13.7**	1.1
	6	0.26	0.02	3.92	0.09	13.37	0.89	**12.3**	1.06
	12	0.2	0.02	3.79	0.10	13.78	0.88	**12.5**	1.1
	48	0.28	0.01	4.99	0.12	14	0.87	**12.8**	1.1
Control HEWL, pH 12.2	1	0.15	0.02	5.06	0.08	14.72	0.90	**13.7**	1.15
	6	0.22	0.01	4.85	0.11	14.58	0.88	**13.4**	1.16
	12	0.78	0.02	5.77	0.14	14.66	0.84	**13.1**	1.04
	48	0.27	0.01	5.42	0.13	14.46	0.86	**13.1**	1.09
